# Time-to-event assessment for the discovery of the proper prognostic value of clinical biomarkers optimized for COVID-19

**DOI:** 10.1016/j.clinsp.2022.100009

**Published:** 2022-02-04

**Authors:** José Raniery Ferreira

**Affiliations:** Hospital das Clínicas HCFMUSP, Faculdade de Medicina, Universidade de São Paulo, São Paulo, SP, Brazil

In the early days of the pandemic, clinical COVID-19 biomarkers were investigated to predict mortality.^1^ Yan et al., for instance, proposed a straightforward decision tree with three variables: Lactic Dehydrogenase (LDH), high-sensitivity C-Reactive Protein (hs-CRP), and lymphocyte percentage. They claimed to obtain more than 90% accuracy on a test set. Although it is an interesting approach, Yan et al. considered the problem a classification task (dead vs. alive), which may not be the proper way to deal with continuous time-to-event data.^2^, ^3^, ^4^ Moreover, machine-learning-based assessment is pruned to over-optimistic results using small sampling for training. In addition, it has been shown that their model has limited performance on external datasets.^5^, ^6^, ^7^ These two limitations are possibly due to data overfitting.

Therefore, the authors performed time-to-event analyses using the original dataset to find a proper predictive potential for the investigated biomarkers. The authors’ evaluation aimed to optimize the clinical variables previously modeled and discover other biomarkers with prognostic value. By opposing the original strategy, the authors also focused on identifying biomarkers for different sub-populations, according to patient aging and hospitalization time.

Original data is publicly available.^1^ The dataset comprised demographics data of age (varying 18–95, averaging 58.8 ± 16.5 years old) and sex (224 men, 151 women), along with the results of 74 blood tests in different hospitalization times. The variables obtained for each patient is listed as follows: 2019-ncov nucleic acid detection, activation of partial thromboplastin time, albumin, alkaline phosphatase, amino-terminal brain natriuretic peptide precursor, antithrombin, aspartate aminotransferase, basophil count, basophil percentage, calcium, corrected calcium, creatinine, d-d dimer, direct bilirubin, egfr, eosinophil count, eosinophils percentage, esr, ferritin, fibrin degradation products, fibrinogen, globulin, glucose, glutamic-pyruvic transaminase, hbsag, hco3-, hcv antibody quantification, hematocrit, hemoglobin, hiv antibody quantification, hypersensitive cardiac troponini, hypersensitive c-reactive protein, indirect bilirubin, interleukin 10, interleukin 1β, interleukin 2 receptor, interleukin 6, interleukin 8, international standard ratio, lactate dehydrogenase, lymphocyte count, lymphocyte percentage, mean corpuscular hemoglobin, mean corpuscular hemoglobin concentration, mean corpuscular volume, mean platelet volume, monocytes count, monocytes percentage, neutrophils count, neutrophils percentage, ph value, platelet count, platelet large cell ratio, plt distribution width, procalcitonin, prothrombin activity, prothrombin time, quantification of treponema pallidum antibodies, rbc distribution width sd, red blood cell count, red blood cell distribution width, serum chloride, serum potassium, serum sodium, thrombin time, thrombocytocrit, total bilirubin, total cholesterol, total protein, tumor necrosis factorα, urea, uric acid, white blood cell count, and γ-glutamyl transpeptidase.

The authors split the dataset into discovery and validation subsets to perform a robust assessment and validate the results. The thresholds identified in the discovery set were then applied in the validation set to confirm further performance. Patient risk groups were stratified according to the variables’ median.^3^^,^^4^ The log-rank test assessed the difference between Kaplan-Meier curves and Cox proportional hazards regression models. R v4.1.0 packages of survival v3.2.3 and survminer v0.4.7 performed statistical analyses, with p < 0.05 considered significant.

As expected, the older the patient is, the worst is the prognosis;^8^^,^^9^ the threshold of 62 years obtained significant difference on survival curves (Fig. 1a). The overall assessment disregarding patient age and hospitalization timing found predictive value in 53 variables, including LDH and hs-CRP (Fig. 1b–c). Moreover, other biomarkers yielded relevant information on COVID-19 prognostication (Table 1). For instance, high-risk groups stratified by fibrin degradation products presented a 97% likelihood of death and a Hazard Ratio (HR) of 4.26 (95% Confidence Interval [95% CI]: 1.88–9.64); and elevated Interleukin-6 (IL-6) associated with 65% likelihood of death and HR of 18.20 (95% CI: 2.42-136.54).

**Fig. 1:**
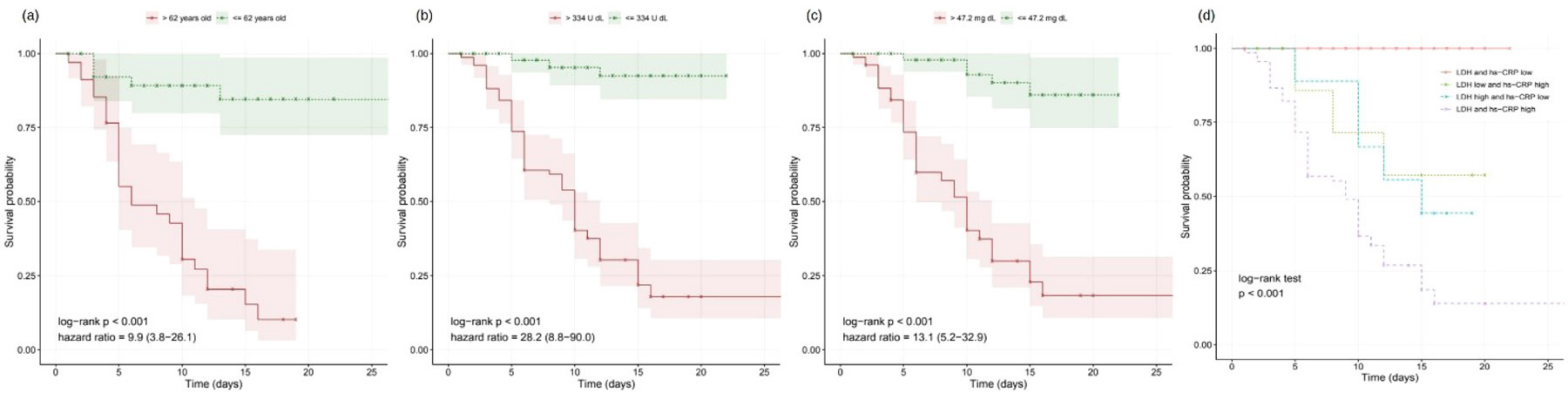
Kaplan-Meier curves of the clinical biomarkers of (a) age, (b) Lactic Dehydrogenase (LDH), (c) high-sensitivity C-Reactive Protein (hs-CRP), (d) LDH combined with hs-CRP.

**Table 1:** Discovered biomarkers according to the patient age and hospitalization time.

			High-risk group		Low-risk group		
	Relevant biomarkers	Threshold discovered	Proportion of deaths	Mean survival days	Proportion of deaths	Mean survival days	Log-rank p-value
**Demographics: 1 significant variable**	Age	62	0.824	7.9	0.146	11.2	< 0.001
	Sex	Male / Female	0.542	9.5	0.296	10.1	0.120
**Overall (disregarding patient age and hospitalization timing): 53 significant variables**	Lactate dehydrogenase	334	0.835	10.1	0.041	12.8	< 0.001
	Hypersensitive c-reactive protein	47.2	0.785	9.6	0.089	13.1	< 0.001
	Lymphocyte (%)	11.6	0.793	10.8	0.134	13.2	< 0.001
	Fibrin degradation products	16.9	0.974	10.4	0.226	10.8	< 0.001
	Interleukin-6	18.3	0.655	10.7	0.045	12.8	< 0.001
	Hypersensitive cardiac troponinI	22.8	0.902	6.8	0.273	12.3	< 0.001
**First sample after admission (disregarding patient age): 40 significant variables**	Lactate dehydrogenase	328	0.732	8.8	0.094	11.2	< 0.001
	Hypersensitive c-reactive protein	51.9	0.732	8.6	0.065	11.6	< 0.001
	Lymphocyte (%)	14.9	0.700	8.8	0.125	11.3	< 0.001
	Fibrin degradation products	4.9	0.875	9.0	0.095	9.6	<0.001
	Interleukin-6	19.53	0.667	10.2	0.071	12.0	< 0.01
	Procalcitonin	0.09	0.853	8.2	0.071	13.6	< 0.001
**Last sample before discharge or death (disregarding patient age): 46 significant variables**	Lactate dehydrogenase	261	0.733	8.6	0.000	11.8	< 0.001
	Hypersensitive c-reactive protein	23.9	0.780	8.0	0.000	12.4	< 0.001
	Lymphocyte (%)	14.35	0.806	8.3	0.083	11.5	< 0.001
	Fibrin degradation products	5.9	0.952	8.7	0.125	9.7	< 0.001
	Procalcitonin	0.09	0.882	8.0	0.036	13.8	< 0.001
	HCO3-	24.1	0.638	7.6	0.115	13.9	< 0.001
**Patients with age <62 years (disregarding hospitalization timing): 31 significant variables**	Lactate dehydrogenase	232.5	0.360	14.1	0.000	18.3	< 0.001
	Hypersensitive c-reactive protein	11.6	0.417	14.1	0.000	18.6	< 0.001
	Lymphocyte (%)	22.15	0.362	15.6	0.089	16.0	< 0.01
	Fibrin degradation products	4	0.769	9.1	0.000	14.4	< 0.001
	International standard ratio	1.05	0.531	11.4	0.000	18.0	< 0.001
	Calcium	2.15	0.463	14.8	0.042	16.2	< 0.001
**Patients with age ≥62 years (disregarding hospitalization timing): 29 significant variables**	Lactate dehydrogenase	470	0.986	11.6	0.603	16.4	< 0.001
	Hypersensitive c-reactive protein	88.3	0.922	12.4	0.596	16.2	< 0.001
	Lymphocyte (%)	5.3	0.967	13.3	0.627	14.2	< 0.01
	Hypersensitive cardiac troponin I	51.4	1.000	11.9	0.800	14.2	< 0.01
	Monocytes (%)	4.1	0.986	13.5	0.552	14.1	< 0.001
	Alkaline phosphatase	77	0.952	11.1	0.687	16.2	< 0.001

Furthermore, LDH and hs-CRP combined presented complementary predictive potential in multivariate assessment (Fig. 1d). With both biomarkers’ values elevated, patients showed a likelihood of death of 87%, the mean survival time of 9.5 days, and HRs of 8.19 (95% CI: 2.27–29.52) and 3.90 (95% CI: 1.41–10.72). Conversely, when either LDH or hs-CRP yielded low value, potentially indicating lower risk, the age determined the worse prognosis in the multivariate signature (p<0.001), resulting in a likelihood of death of 72% and HR of 7.01 (95% CI: 3.10–15.84) for the elderly patients.

Results confirmed poor short-term prognosis to abnormal levels of some indicators, such as LDH,^1^^,^^9^, ^10^, ^11^ CRP,^1^^,^^8^, ^9^, ^10^, ^11^ lymphocytes,^1^^,^^8^, ^9^, ^10^ IL-6,^12^ and procalcitonin.^11^ These findings could provide insights into COVID-19 research, such as key levels of fibrin degradation products, which are directly associated with the Dimerized plasmin fragment D and could indicate active coagulation and thrombosis.^9^, ^10^, ^11^

Yan et al. had already mentioned that lymphocytes might serve as a potential therapeutic target.^1^ Still, the authors highlight the role of IL-6, a cytokine that induces inflammatory response and has prognostic value. Although IL-6 blockade is not the standard strategy for COVID-19 treatment, interleukin-6 remains the best available biomarker for severity assessment and still holds great potential for targeted therapy.^12^

In this work, the authors have identified relevant biomarkers that are fully available in medical practice and be a mainstay for the clinical evaluation of COVID-19. These biomarkers correlated with short-term outcomes and could support the management of the disease with early interventions, ultimately leading to better endpoints such as decreased deterioration and mortality. Future works include a prospective evaluation to increase robustness and the assessment across different geographic populations, as each region has its genomic specificity.

## Conflicts of interest

The authors declare no conflicts of interest.
